# Supplementary material for: Phosphoribosyl ubiquitination of SNARE proteins regulates autophagy during Legionella infection
Source: EMBO J. 2025 Jun 12;44(15):4252–79. doi: 10.1038/s44318-025-00483-4 (PMC12317070; doi:10.1038/s44318-025-00483-4)
Supplement: Supplementary file 7 — Expanded View Figures [file 44318_2025_483_MOESM7_ESM.pdf]

Expanded View Figures

Figure EV1. PR-Ub regulates autophagy in Legionella infection.

(**A**) SNARE proteins that were identified as PR-Ub substrates (Shin et al, 2020). (**B**) A549 cells were infected with different strains of *Legionella* for 2 h in the presence of 300 nM bafilomycin A1, then lysed and analyzed by western blot with antibodies against LC3 (and GAPDH as a loading control). The experiment was repeated three times with similar results. (**C**) HEK 293T cells were transfected with SdeA, its catalytic mutant SdeA (EE/AA) or a control vector for 16 h. Cells were treated with 100 nM Torin-1 as shown, then lysed and analyzed by western blot to check for LC3 levels. GAPDH was used as a loading control. Graph represents data from three independent experiments. Error bars indicate standard deviation. *P* value was calculated using two-tailed, type 3 Student's *t* test, **0.01 ≤ $P < 0.001$. (**D**) HeLa cells were cotransfected with RFP-GFP-LC3 and HA-tagged SdeA/SdeA(EE/AA) or a control vector for 16 h before treatment with 300 nM Torin-1 for 4 h to induce autophagy. The cells were then fixed and incubated with an anti-HA antibody for confocal imaging. We counted the total number of puncta and the number of red puncta per cell in FIJI. The data are means ± SEM of 30 cells from three independent experiments (**$P = 0.002$ (vector vs SdeA), **$P = 0.041$(SdeA vs SdeA(EE/AA)). Scale bar: 5 µm. Dotted lines indicate cell outlines drawn from thresholding images in FIJI. (**E**) A549 cells were infected with the indicated strains of *Legionella*, and their intracellular replication was assessed at 0, 24 and 48 h post-infection. The data are means ± SEM of three independent experiments *$P = 0.012$ (WT vs ΔS), **$P = 0.00203$ (WT vs ΔRΔS). (**F**) RAW264.7 cells were infected with the indicated strains of *Legionella*, and their intracellular replication was assessed at 0, 24 and 48 h post-infection. The data are means ± SEM of three independent experiments. **$P = 0.0062$ (WT vs ΔS), **$P = 0.00072$ (WT vs ΔRΔS).

© The Author(s)

(A)

Majority protein ID	Gene names	log2 enrichment factor dDup12 over dSidE	-Log10 p-value dDup12 over dSidE	log2 enrichment factor WT over dSidE	-Log10 p-value WT over dSidE
O00161	SNAP23	4,29	2,41	3,94	2,21
O60749	SNX2	3,72	2,87	3,97	3,17
P56962	STX17	2,39	3,17	2,47	3,45
Q9UNK0	STX8	2,07	2,28	2,18	2,28
O95721	SNAP29	2,96	3,27	0,94	1,89
Q9UNH7	SNX6	1,83	2,08	2,17	2,08
Q13190	STX5	1,60	1,64	1,72	1,83

(B)

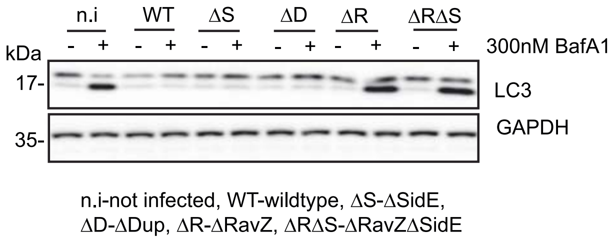

(C)

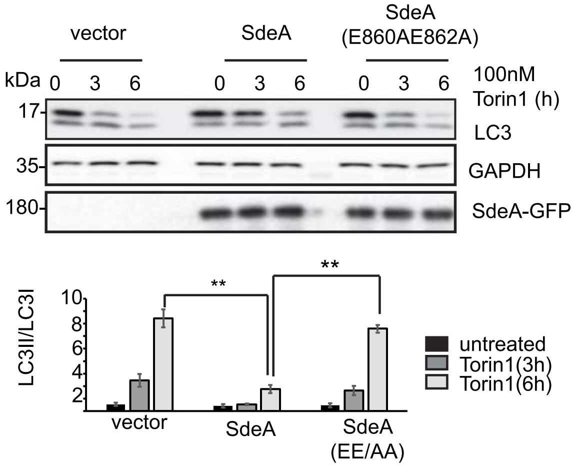

(D)

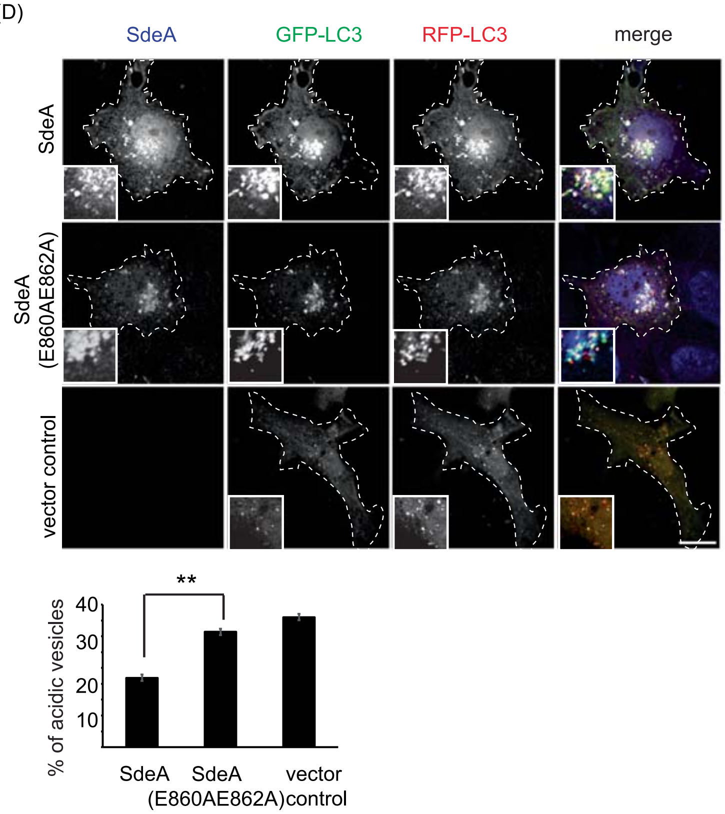

(E)

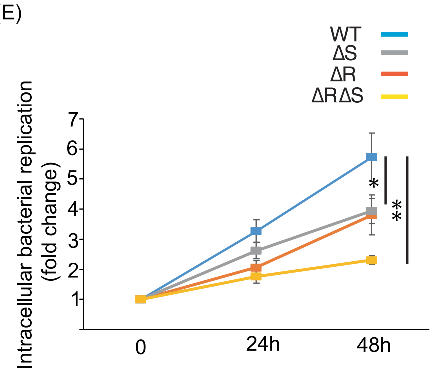

(F)

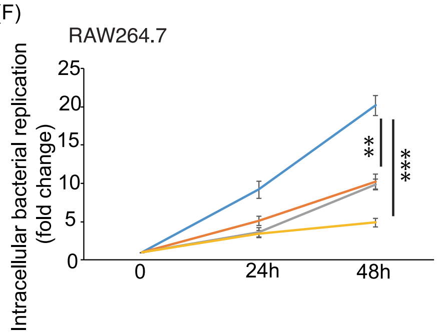

 © The Author(s)

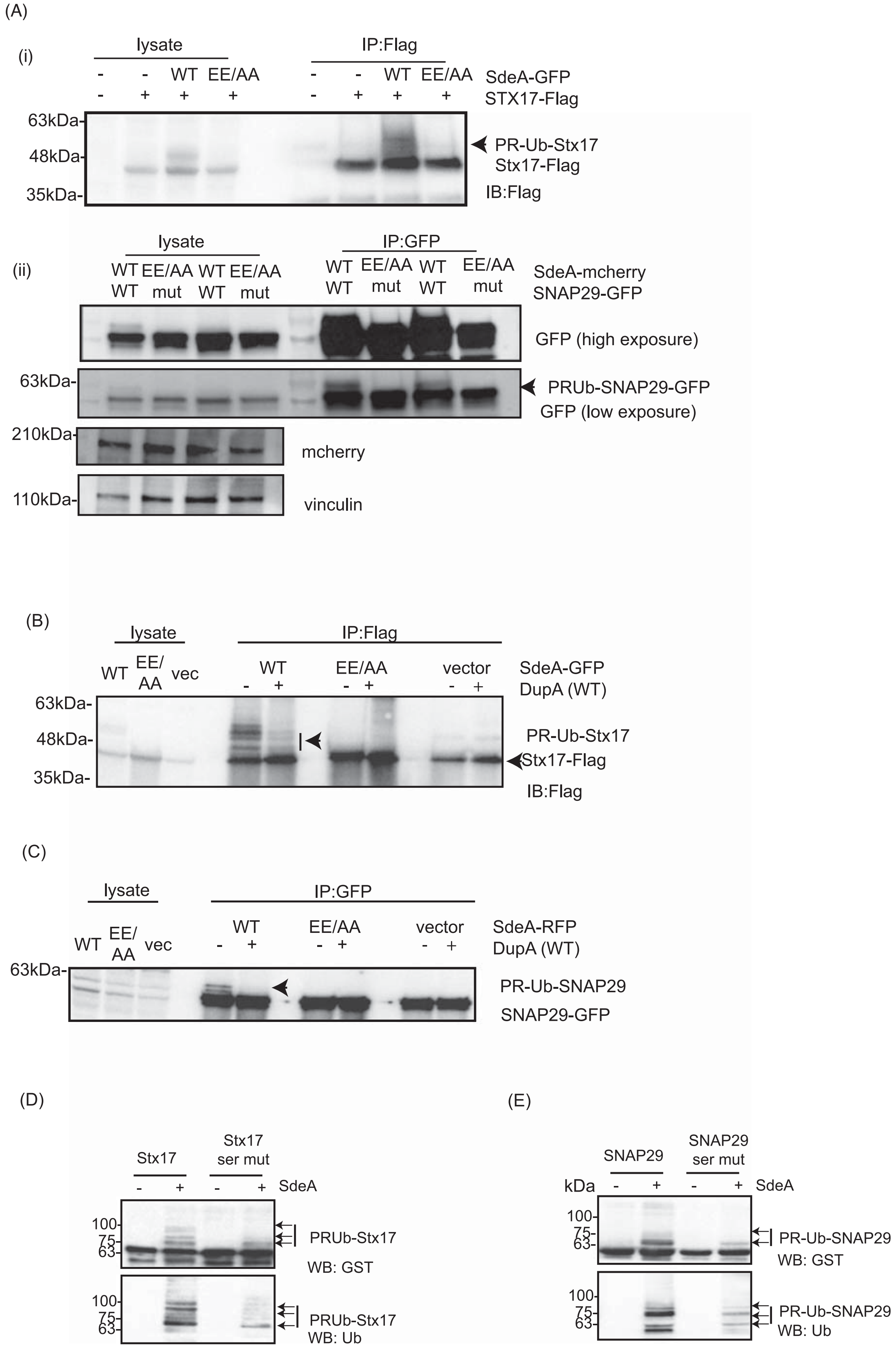
(A)
(i)
lysate
IP:Flag
- - WT EE/AA - - WT EE/AA SdeA-GFP
- + + + - + + + STX17-Flag
63kDa-
48kDa-
35kDa-
PR-Ub-Stx17
Stx17-Flag
IB:Flag
(ii)
lysate
IP:GFP
WT EE/AA WT EE/AA WT EE/AA WT EE/AA SdeA-mcherry
WT mut WT mut WT mut WT mut SNAP29-GFP
GFP (high exposure)
63kDa-
PRUb-SNAP29-GFP
GFP (low exposure)
210kDa-
mcherry
110kDa-
vinculin
(B)
lysate
IP:Flag
WT EE/AA vec
WT EE/AA vector SdeA-GFP
- + - + - + DupA (WT)
63kDa-
48kDa-
35kDa-
PR-Ub-Stx17
Stx17-Flag
IB:Flag
(C)
lysate
IP:GFP
WT EE/AA vec
WT EE/AA vector SdeA-RFP
- + - + - + DupA (WT)
63kDa-
PR-Ub-SNAP29
SNAP29-GFP
(D)
Stx17
Stx17 ser mut
- + - + SdeA
100-
75-
63-
PRUb-Stx17
WB: GST
PRUb-Stx17
WB: Ub
(E)
SNAP29
SNAP29 ser mut
kDa - + - + SdeA
100-
75-
63-
PR-Ub-SNAP29
WB: GST
PR-Ub-SNAP29
WB: Ub

Figure EV2. STX17 and SNAP29 are modified by PR-Ub on specific serine residues during Legionella infection.

(**A**) (i) HEK 293 T cells were cotransfected with FLAG-STX17 and GFP-tagged SdeA/SdeA(EE/AA) or a control vector for 16 h. FLAG-STX17 was immunoprecipitated using FLAG resin and analyzed by western blot using antibodies against FLAG to detect PR-Ub-modified and unmodified FLAG-STX17. The experiment was repeated 2 times with similar results. (ii) HEK 293T cells were cotransfected with FLAG-STX17 and GFP-tagged SdeA/SdeA(EE/AA) or a control vector and immunoprecipitated as shown in (**B**). The samples were then treated with or without pure DupA for 1 h before western blotting with antibodies against FLAG to detect PR-Ub-modified and unmodified FLAG-STX17. The experiment was repeated two times with similar results. (**B**) HEK 293T cells were cotransfected with GFP-SNAP29 and HA-tagged SdeA/SdeA(EE/AA) or a control vector for 16 h. GFP-SNAP29 was immunoprecipitated with anti-GFP beads, treated with or without pure DupA for 1 h and analyzed by western blot with antibodies against GFP to detect PR-Ub-modified and unmodified SNAP29. The experiment was repeated two times with similar results. (**C**) GST-STX17 and GST-STX17(S195AS202AS209A) were incubated with or without SdeA in the presence of 1 mM NAD^+ and ubiquitin for 1 h. The samples were analyzed by western blot using antibodies against ubiquitin and GST to detect PR-Ub. The experiment was repeated three times with similar results. (**D**) GST-STX17 and its PR-Ub-deficient mutant (S195AS202AS209A) were modified with or without SdeA, in the presence of 1 mM NAD+ and ubiquitin for 1 h. Samples were analyzed by western blot with antibodies against ubiquitin and GST to detect PR-Ub. The experiment was repeated three times with similar results. SdeA(EE/AA): mART mutant SdeA(E860AS862A) (**E**) GST-SNAP29 and its PR-Ub-deficient mutant (S61AS63AS70A) were modified with or without SdeA, in the presence of 1 mM NAD^+ and ubiquitin for 1 h. Samples were analyzed by western blot with antibodies against ubiquitin and GST to detect PR-Ub. The experiment was repeated three times with similar results. SdeA(EE/AA): mART mutant SdeA(E860AS862A).

 © The Author(s)

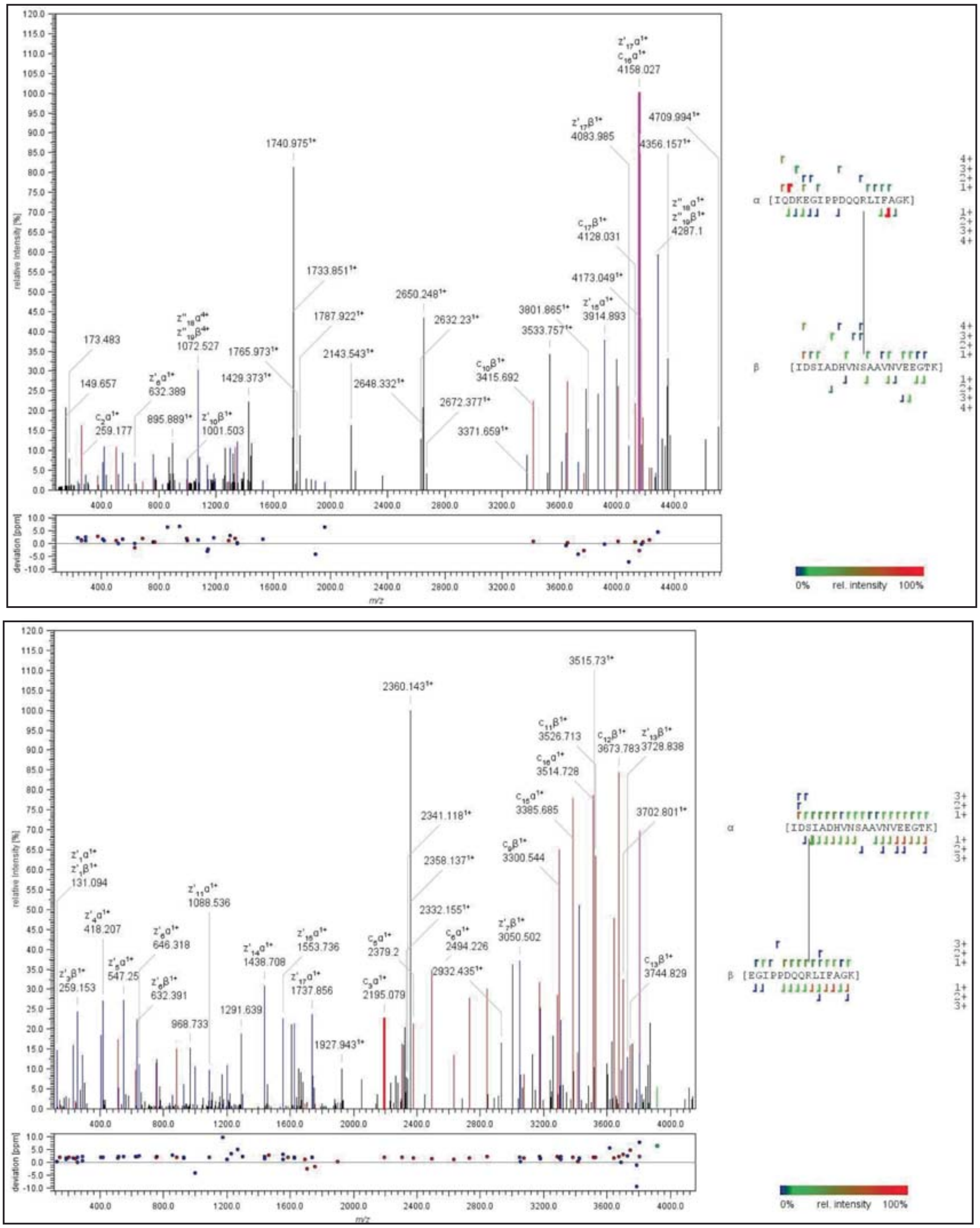

MS spectra of PR-Ub modified STX17(1-224)

Figure EV3. Identification of STX17 residues modified by PR-Ub.

Mass spectra of PR-Ub-modified STX17(1–224). S202, S209 were identified as the modified serine residues by high-resolution ETD mass spectrometry.

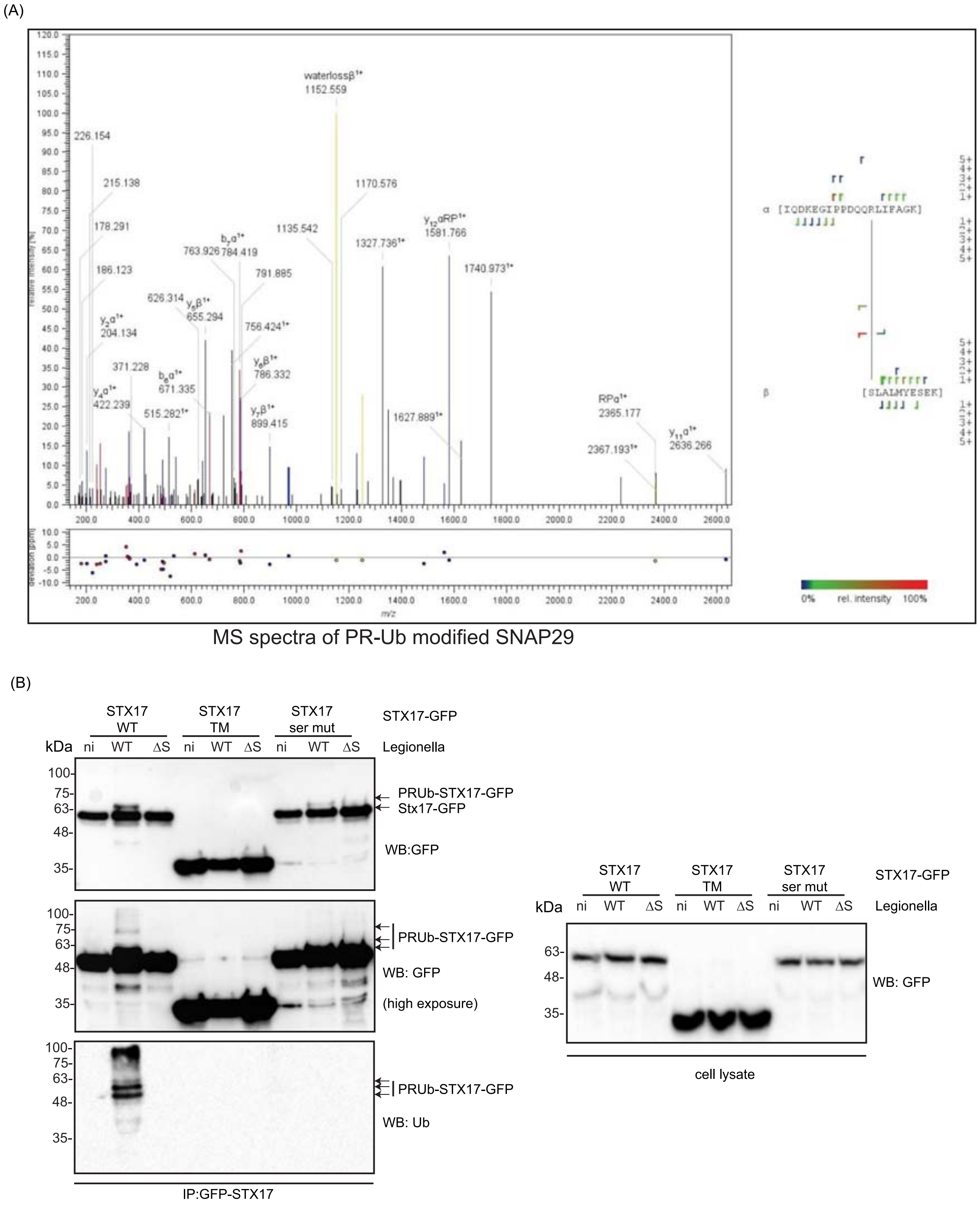

Figure EV4. Identification and validation of serine residues on STX17 and SNAP29 that are modified by PR-Ub.

(**A**) Mass spectrum and deduced sequence map of PR-Ub-modified SNAP29. (**B**) HEK 293 T cells were transfected with GFP-tagged WT STX17, STX17TM, or the STX17 serine mutant (S195AS202AS209A) followed by *Legionella* infection for 2 h. STX17 was then immunoprecipitated using anti-GFP beads followed by western blotting with antibodies against GFP and ubiquitin. Cell lysates were analyzed by western blot with an antibody against GFP to check the expression levels of GFP-STX17 constructs. The experiment was repeated three times with similar results.

 © The Author(s)

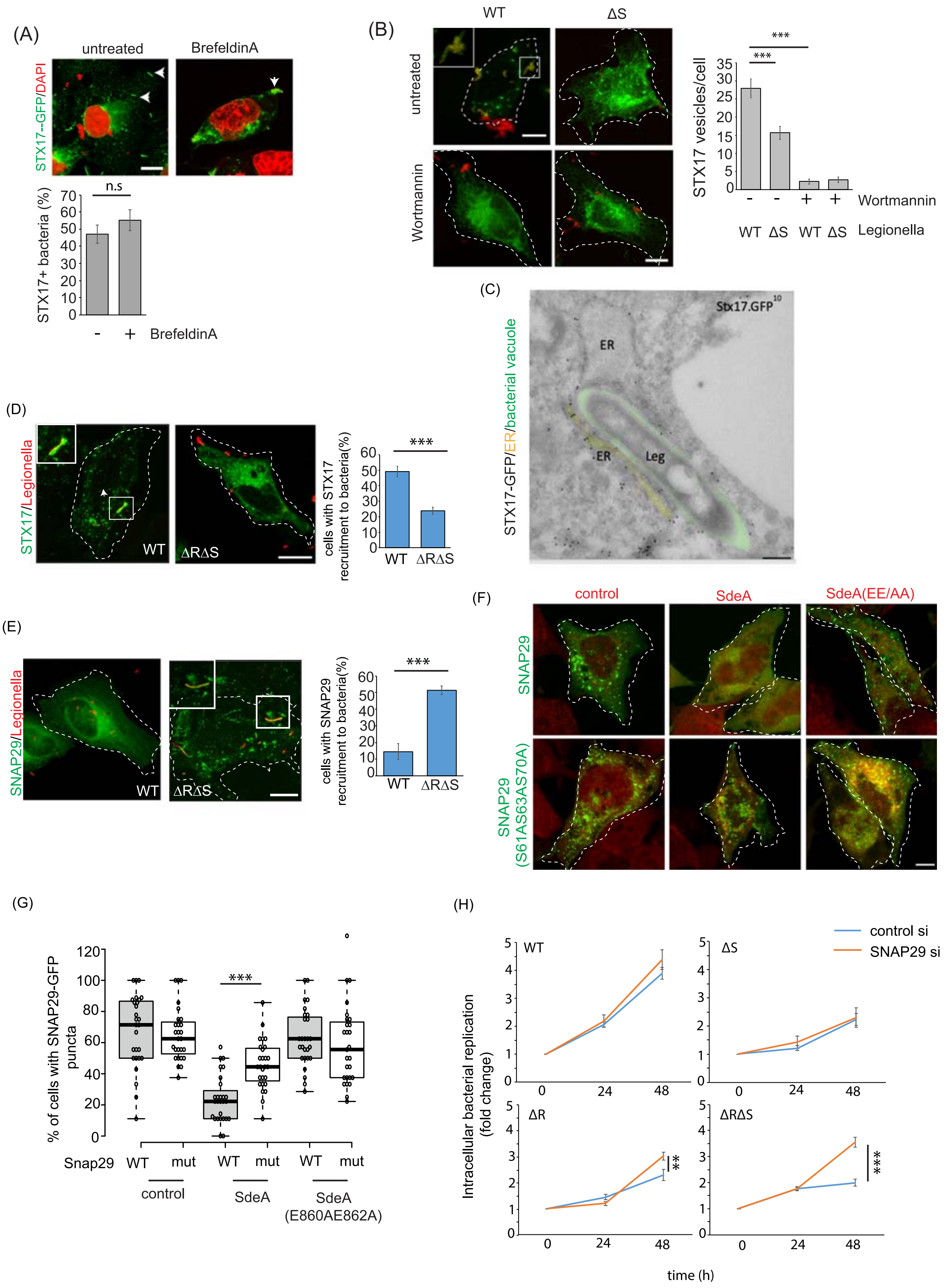
(A)
untreated
BrefeldinA
STX17--GFP/DAPI
n.s
STX17+ bacteria (%)
BrefeldinA
(B)
WT
ΔS
untreated
Wortmannin
***
STX17 vesicles/cell
Wortmannin
WT ΔS WT ΔS Legionella
(C)
STX17-GFP/ER/bacterial vacuole
Stx17.GFP
ER
Leg
(D)
STX17/Legionella
WT
ΔRΔS
cells with STX17 recruitment to bacteria(%)
(E)
SNAP29/Legionella
WT
ΔRΔS
cells with SNAP29 recruitment to bacteria(%)
(F)
control
SdeA
SdeA(EE/AA)
SNAP29
SNAP29 (S61AS63AS70A)
(G)
% of cells with SNAP29-GFP puncta
Snap29 WT mut WT mut WT mut
control
SdeA
SdeA (E860AE862A)
(H)
WT
ΔS
ΔR
ΔRΔS
control si
SNAP29 si
Intracellular bacterial replication (fold change)
time (h)

Figure EV5. Recruitment of STX17 and SNAP29 to bacterial vacuoles is regulated by their PR-Ub.

(**A**) A549 cells expressing STX17-GFP were infected with WT *Legionella* for 2 h in the presence or absence of 100 nM brefeldin A before fixation and staining the intracellular bacteria by DAPI. STX17-positive bacteria was counted in 50 cells per set, taken from three independent experiments. Error bars indicate SEM. Difference between sets was non-significant from p value calculated by two-tailed, type 3 Student's *t* test. Scale bar:10 µm. (**B**) A549 cells expressing STX17-GFP were infected with *Legionella* (WT/ΔS) for 2 h in the presence or absence of 100 nM wortmannin before fixation and immunostaining with antibodies against *Legionella*. p value was calculated by two-tailed, type 3 Student's *t* test. ***$P = 4.45E\text{-}6$(WT and ΔS sets without wortmannin), ***$P = 2.05E\text{-}5$ (WT +/-wortmannin), Graph represents $n = 50$ cells taken from three experiments, error bars indicate SEM. Scale bar: 5 µm. Dotted lines indicate cell outlines drawn from thresholding images in FIJI. (**C**) Immuno-electron microscopy of HeLa cells transfected with STX17-GFP and infected with WT Legionella-DsRed for 4 h. Ultrathin cryosection immunogold labeled for STX17-GFP by protein A–10-nm gold. Colors are added by Photoshop: Yellow marks a STX-17.GFP-positive ER cisterna closely aligned with the Legionella (Leg) containing vacuole. Green marks the space between the vacuolar membrane and enclosed Legionella. Bar, 200 nm. (**D**) A549 cells were infected with WT or ΔRΔS *Legionella* for 1 h, fixed and immunostained with the STX17 and Legionella antibodies to check for the recruitment of STX17 to intracellular bacteria. White arrows mark intracellular bacteria with STX17 recruitment. The data are means ± SEM of 118 cells from three independent experiments. p value was calculated by two-tailed, type 3 Student's *t* test. ***$P = 4.21E\text{-}6$. Scale bar: 5 µm. Dotted lines indicate cell outlines drawn from thresholding images in FIJI. (**E**) A549 cells were infected with WT or ΔRΔS *Legionella* for 1 h, fixed and immunostained with the SNAP29 and Legionella antibodies to check for the recruitment of SNAP29 to intracellular bacteria. White arrows mark intracellular bacteria with SNAP29 recruitment. The data are means ± SEM of 120 cells from three independent experiments. p value was calculated by two-tailed, type 3 Student's *t* test. ***$P = 2.21E\text{-}4$. Scale bar: 5 µm. Dotted lines indicate cell outlines drawn from thresholding images in FIJI. (**F**) HeLa cells were cotransfected with RFP-tagged SdeA or its catalytic mutant (E860AE862A) and GFP-tagged WT SNAP29 or its PR-Ub-deficient mutant. Cells were treated with 300 nM Torin-1 for 4 h to induce autophagy before fixation and confocal imaging. Scale bar:5 µm. Dotted lines indicate cell outlines drawn from thresholding images in FIJI. (**G**) The graph shows the number of cells with SNAP29-GFP puncta (from panel d) counted in FIJI. In the box plot, center lines show the medians; box limits indicate the 25th and 75th percentiles as determined by R software; whiskers extend 1.5 times the interquartile range from the 25th and 75th percentiles. n > 30 cells taken from three independent experiments. *P* value was calculated using two-tailed, type 3 Student's *t* test, ***$P = 0.00032$. In bar graph, the data are means ± SEM of n > 30 cells from three independent experiments. Scale bar:5 µm. (**H**) A549 cells were treated with SNAP29 or control siRNA for 48 h followed by infection Legionella. Intracellular bacterial replication was assessed after 0, 24 and 48 h. Data are means ± SEM of three independent experiments. *P* value was calculated using two-tailed, type 3 Student's *t* test, ***$P = 2.1E\text{-}4$ (ΔR), **$P = 0,.031$(ΔRΔS). (ni not infected, WT wild-type, *Legionella*, ΔS-ΔSidE *Legionella*).

 © The Author(s)

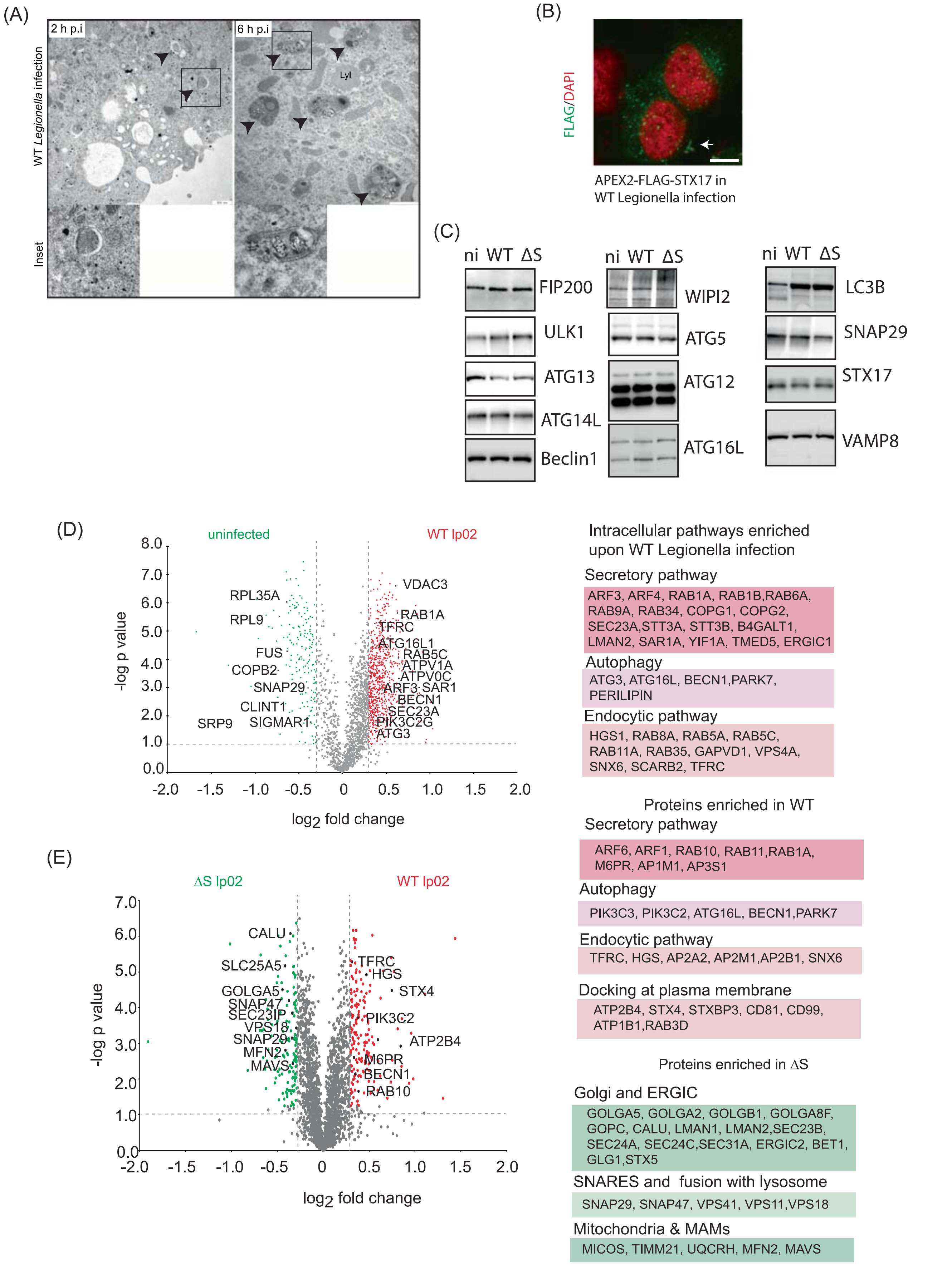
(A)
2 h p.i
6 h p.i
Lyl
WT Legionella infection
Inset
(B)
FLAG/DAPI
APEX2-FLAG-STX17 in
WT Legionella infection
(C)
ni WT ΔS
FIP200
ULK1
ATG13
ATG14L
Beclin1
WIPI2
ATG5
ATG12
ATG16L
LC3B
SNAP29
STX17
VAMP8
(D)
uninfected
WT lp02
-log p value
log2 fold change
RPL35A
RPL9
FUS
COPB2
SNAP29
CLINT1
SRP9
SIGMAR1
VDAC3
RAB1A
TFRC
ATG16L1
RAB5C
ATPV1A
ATPV0C
ARF3 SAR1
BECN1
SEC23A
PIK3C2G
ATG3
Intracellular pathways enriched
upon WT Legionella infection
Secretory pathway
ARF3, ARF4, RAB1A, RAB1B,RAB6A, RAB9A, RAB34, COPG1, COPG2, SEC23A,STT3A, STT3B, B4GALT1, LMAN2, SAR1A, YIF1A, TMED5, ERGIC1
Autophagy
ATG3, ATG16L, BECN1,PARK7, PERILIPIN
Endocytic pathway
HGS1, RAB8A, RAB5A, RAB5C, RAB11A, RAB35, GAPVD1, VPS4A, SNX6, SCARB2, TFRC
(E)
ΔS lp02
WT lp02
-log p value
log2 fold change
CALU
SLC25A5
GOLGA5
SNAP47
SEC23IP
VPS18
SNAP29
MFN2
MAVS
TFRC
HGS
STX4
PIK3C2
ATP2B4
M6PR
BECN1
RAB10
Proteins enriched in WT
Secretory pathway
ARF6, ARF1, RAB10, RAB11,RAB1A, M6PR, AP1M1, AP3S1
Autophagy
PIK3C3, PIK3C2, ATG16L, BECN1,PARK7
Endocytic pathway
TFRC, HGS, AP2A2, AP2M1,AP2B1, SNX6
Docking at plasma membrane
ATP2B4, STX4, STXBP3, CD81, CD99, ATP1B1,RAB3D
Proteins enriched in ΔS
Golgi and ERGIC
GOLGA5, GOLGA2, GOLGB1, GOLGA8F, GOPC, CALU, LMAN1, LMAN2,SEC23B, SEC24A, SEC24C,SEC31A, ERGIC2, BET1, GLG1,STX5
SNARES and fusion with lysosome
SNAP29, SNAP47, VPS41, VPS11,VPS18
Mitochondria & MAMs
MICOS, TIMM21, UQCRH, MFN2, MAVS

© The Author(s)

Figure EV6. Proximity labeling of STX17 during *Legionella* infection.

(**A**) HeLa cells expressing CD32 (for efficient uptake of *Legionella*) were infected with WT Legionella for 2 h or 6 h. Cells were fixed using 2.5% glutaraldehyde in 0.1 M cacodylate buffer for two hours at RT. Cells were scraped of the petridish, post-fixed with 1% reduced osmium tetroxide at RT, dehydrated and embedded using EPON. Ultrathin sections (50 nm) were imaged by transmission electron microscopy. Arrows mark intracellular bacteria, Lyl-lysosome-like organelles. (**B**) HeLa cells expressing APEX2-FLAG-STX17 are infected with WT *Legionella* for 2 h followed by fixing cells and staining cells with FLAG antibody to check its recruitment to intracellular bacteria. DAPI marks nuclear DNA and cytosolic bacteria. Scale bar:5 µm. White arrows indicate intracellular bacteria. (**C**) Lysates used as input in streptavidin IP shown in Fig. 4G. (**D**) Volcano plot showing how the biotin-labeled proteome changes when HeLa cells expressing APEX-STX17 are infected with WT *Legionella* for 2 h; GO analysis of the biotin-labeled proteome showing pathways upregulated by infection with WT *Legionella*. Red and green indicate compartments containing proteins enriched following infection with WT *Legionella* and in uninfected cells, respectively. Data represent mean fold change of three experimental replicates per infection set ($n = 3$). *P* value was calculated using two-tailed type 3 Student's *t* test and significant candidates were chosen having *P* value ≤ 0.01 and log2(fold change) value minimum of ±0.5. (**E**) Volcano plot showing changes in the biotin-labeled proteome following the infection of HeLa cells expressing APEX-STX17 with WT and ΔS *Legionella* for 2 h. GO analysis of the biotin-labeled proteome showing pathways upregulated by infection with WT vs ΔS *Legionella*. Data represents mean fold change of three experimental replicates per infection set ($n = 3$). *P* value was calculated using two-tailed type 3 Student's *t* test and significant candidates were chosen having *P* value ≤ 0.01 and log2 (fold change) value minimum of ±0.5. Red and green indicate compartments containing proteins enriched following infection with WT and ΔS *Legionella*, respectively. (ni not infected, WT wild-type *Legionella*, ΔS-ΔSidE).

 © The Author(s)
